# Case Report: Insulin hypersensitivity in youth with type 1 diabetes

**DOI:** 10.3389/fendo.2023.1226231

**Published:** 2023-10-20

**Authors:** Einas H. Alkhatib, Jody B. Grundman, Anna M. Adamusiak, Melena D. Bellin, Joel P. Brooks, Kevin S. Buckley, Erin M. Janssen, Maleewan Kitcharoensakkul, Kyle P. McNerney, Thea L. Pfeifer, Brooke I. Polk, Brynn E. Marks

**Affiliations:** ^1^ Department of Pediatric Endocrinology, Children’s National Hospital, Washington, DC, United States; ^2^ Department of Surgery, University of Minnesota, Minneapolis, MN, United States; ^3^ Department of Pediatrics, Division of Endocrinology, University of Minnesota, Minneapolis, MN, United States; ^4^ Department of Allergy and Immunology, Columbia University/New York-Presbyterian, New York, NY, United States; ^5^ Departments of Hematology/Oncology and Infectious Disease, Atrium Health Levine Children’s Hospital, Concord, NC, United States; ^6^ Department of Rheumatology, Mott Children’s Hospital/University of Michigan, Ann Arbor, MI, United States; ^7^ Departments of Pediatric Allergy and Pulmonary Medicine, Washington University School of Medicine, St. Louis, MO, United States; ^8^ Department of Pediatric Endocrinology, Washington University School of Medicine, St. Louis, MO, United States; ^9^ Department of Pediatric Endocrinology, Atrium Health Levine Children’s Hospital, Concord, NC, United States; ^10^ Department of Endocrinology and Diabetes, Children’s Hospital of Philadelphia, Philadelphia, PA, United States

**Keywords:** insulin hypersensitivity, type 1 diabetes, pediatrics, pancreas transplant, insulin allergy

## Abstract

**Objective:**

Immediate type I, type III, and delayed type IV hypersensitivity reactions to insulin are rare, but potentially serious complications of exogenous insulin administration required for the treatment of type 1 diabetes (T1D).

**Methods:**

We present four cases of insulin hypersensitivity reactions occurring in youth with T1D and a literature review of this topic.

**Results:**

Insulin hypersensitivity reactions included types I, III, and IV with presentations ranging from localized urticaria, erythematous nodules, and eczematous plaques to anaphylaxis with respiratory distress. Reactions occurred in youth with newly diagnosed T1D and in those with long-standing T1D who were using both injection and insulin pump therapy. Multidisciplinary care involving pediatric endocrinology and allergy/immunology utilizing trials of many adjunct therapies yielded minimal improvement. Despite the use of various treatments, including antihistamines, topical therapies, immunosuppressant medications, desensitization trials, and intravenous immune globulin, cutaneous reactions, elevated hemoglobin A1c levels, and negative effects on quality of life remain persistent challenges. One patient became one of the youngest pancreas transplant recipients in the world at age 12 years due to uncontrollable symptoms and intolerable adverse effects of attempted therapies.

**Conclusion:**

Although rare, insulin hypersensitivity reactions negatively affect glycemic control and quality of life. These cases demonstrate the varying severity and presentation of insulin hypersensitivity reactions along with the limited success of various treatment approaches. Given the life-sustaining nature of insulin therapy, further studies are needed to better understand the underlying pathophysiology of insulin hypersensitivity and to develop targeted treatment approaches.

## Introduction

Type I, type III, and type IV insulin hypersensitivity reactions are rare, but potentially serious complications of insulin treatment of type 1 diabetes (T1D). Type I, IgE-mediated reactions often present within seconds to minutes as localized urticaria. More rarely, they may be delayed by several hours or present with anaphylaxis. Type III, antigen–antibody complex-mediated reactions often present as localized Arthus reactions within 24 h with painful subcutaneous nodules. However, they can present 4–10 days later as generalized serum sickness reactions. Delayed, type IV hypersensitivity reactions are triggered by T-cell activation and may present within days as contact dermatitis with eczematous, erythematous areas (1, 2).

Insulin reactions can be refractory to treatment leading to poor glycemic control and quality of life (1, 2). Although there are several reported adult cases (3–6), data in children are limited. We present four pediatric cases of insulin hypersensitivity reactions, including one of the youngest pancreas transplant recipients at the age of 12 years. These cases were identified at four pediatric hospitals, which collectively treat approximately 7,100 patients with T1D, between 2016 and 2023.

## Case A

Patient A is an 18-year-old female adolescent, diagnosed with T1D at age 4 years. She was initially managed exclusively with lispro given a low hemoglobin A1c (HbA1c) of 7.6%. Three days later, upon discharge, she developed a type I, IgE-mediated hypersensitivity reaction with respiratory distress when glargine was added (**Table 1**), prompting intramuscular epinephrine administration and an emergency room visit. She was transitioned to detemir but developed type IV, localized skin reactions within days. After 3 weeks, similar localized eczematous plaques to glulisine developed. The localized reactions were partially abated by oral diphenhydramine. Allergy evaluation and reports of prior anaphylaxis to pickles and whipped cream identified the suspected trigger as polysorbate 20, which is common to glargine, glulisine, and the aforementioned foods. She was transitioned to lispro, which does not contain polysorbate 20. However, localized eczematous-like reactions persisted, and increasing insulin doses were required. This raised concern for a meta-cresol allergy since it is found in all insulins. However, patch testing was negative. She was switched to aspart, which was better tolerated despite containing meta-cresol, but the localized skin reactions persisted.

**Table 1:** Summary of insulin hypersensitivity cases.

Patient	Current Age (years)	T1D Onset (years)	Duration of T1D at Reaction Onset	Presenting Symptoms	Hypersensitivity Reaction Type	Current Management	Total Daily Insulin Dose (units/kg/day)	Most Recent HbA1c
A	18	4	Within days of diagnosis	Anaphylaxis^*1^, with respiratory distress - Generalized urticaria	-Type I with anaphylaxis -Type IV, possibly due to polysorbate 20	- 4.75 years s/p pancreas transplant - Tacrolimus - Everolimus - Prednisone	Not currently requiring insulin therapy	5.3%
B	16	7	2 years	-Bruising -Erythema -Leg pain	- Type III	Subcutaneous insulin injections Lispro, Detemir - Methotrexate - IVIG - Rituximab	2.26	9.5%
C	8	1.2	1.8 years	Eczematous, erythematous plaques	Type IV, possibly due to acrylate	- Aspart via insulin pump -Methotrexate	0.67	8.3%
D	15	2	6 years	Erythematous, firm, tender nodules	Type III vs. IV	Aspart via insulin pump (site changes every 36–48 h)	1.7	9.0%

*1NIAID/FAAN anaphylaxis criteria met with a Brighton Collaboration score of 1 (**Supplementary Data Sheet 1**).

**Figure 1:**
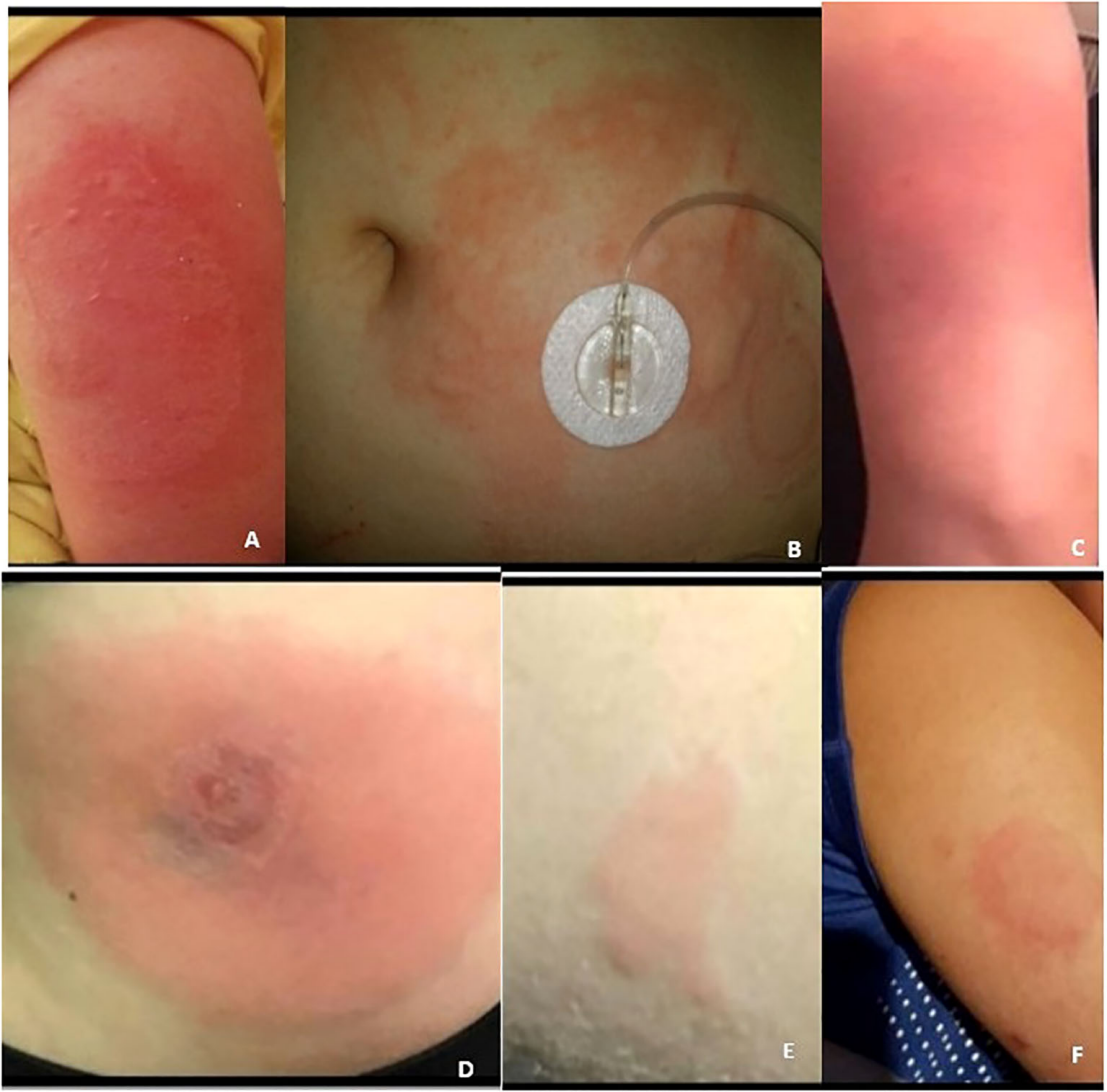
**(A–F)** Patients **(A–D)** hypersensitivity reactions.

Patient A trialed a Tandem T:Slim insulin pump in an effort to decrease reactions by continuously administering small insulin doses. She developed large local reactions with Teflon pump cannulas (**Figures 1A, B**), which improved modestly with a stainless-steel cannula. She was simultaneously trialed on mycophenolate and prednisone but developed infusion site cellulitis and bacteremia. These immunosuppressants also led to cataracts and peripheral neuropathy. High-dose antihistamines led to intolerable fatigue, and reactions persisted. By age 11, she required a total daily insulin dose (TDD) of 1.26 units/kg/day and hemoglobin A1c’s ranged from 7.2% to 7.7%.

Intravenous (IV) insulin desensitization was trialed. Although she had significant discomfort and localized, eczematous reactions with peripheral insulin infusions, central infusions were better tolerated. Ultimately, these localized reactions persisted and desensitization was deemed unsuccessful.

After 8 years, she was referred for pancreas transplant. In February 2018, at age 12, she became one of the youngest pancreas transplant recipients to date. Despite two episodes of rejection at 1 and 14 months post-transplant, pancreatic autoantibodies remain negative. There was no evidence of endocrine pancreatic failure during the acute rejection episodes. Though it has not been required, a plan was formulated to pursue a preservative-free insulin through special manufacturing and a single patient compassionate investigational new drug (IND) use through the Food and Drug Administration (FDA). She remains euglycemic off insulin therapy nearly 5 years post-transplant. The immunosuppressant medications include tacrolimus, everolimus, and prednisone (15.8 mg/m^2^/day of hydrocortisone equivalent). She has transiently required insulin to correct for hyperglycemia while on high-dose glucocorticoids for acute rejection management, which was well tolerated after pre-treatment with diphenhydramine and anti-thymocyte globulin. Two years post-transplant, she developed pancreatitis with no known trigger. Pancreatic enzyme supplementation was initiated for possible pancreatic insufficiency.

At the most recent endocrinology follow-up 4.75 years post-transplant, body mass index (BMI) was in the 97th percentile with fasting glucose 100 mg/dL and HbA1c 5.3%. Metformin was incrementally increased from 500 mg to 2,000 mg daily, then decreased to 1,000 mg daily due to abdominal discomfort.

In case of mild hyperglycemia and despite a prior episode of pancreatitis, a glucagon-like peptide-1 receptor agonist will be considered, paired with a proton pump inhibitor to stimulate beta cell neogenesis and gastrin-mediated glycemic improvement (7). If severe hyperglycemia develops, a peripherally inserted central catheter (PICC) line would be placed for an aspart infusion at 0.03 units/kg/h, titrating glucoses to 150–200 mg/dL.

## Case B

Patient B is a 16-year-old female adolescent, diagnosed with T1D at age 7 years. She tolerated insulin injections with lispro and glargine for 2 years, but within 6 weeks of initiating Tandem T:Slim insulin pump therapy with lispro, she developed suspected type III hypersensitivity reactions with localized erythema and swelling (**Figures 1C, D**). Pump use was discontinued in favor of multiple daily injection therapy. Aspart, lispro, glulisine, glargine, and detemir injections were trialed, but localized, large, painful, erythematous skin lesions occurred. Pump therapy was trialed again 1.5 years later, but the reactions remained unchanged.

She was admitted for evaluation by endocrinology, dermatology, allergy/immunology, and pharmacy. Skin biopsy demonstrated extravasated red cells suggestive of type III hypersensitivity. However, type I hypersensitivity features included eosinophils, mast cells, and spongiosis. Alternate complement (AH50), classical complement (CH50), immune complex assay, insulin antibody, and insulin IgE laboratory studies were normal. Injections with neutral protamine Hagedorn (NPH), regular insulin, lispro, and detemir yielded similar reactions. Adjuvant systemic prednisone, cetirizine 10 mg twice daily, ranitidine 150 mg twice daily, montelukast, topical steroids, and tacrolimus were trialed. High-dose prednisone (100 mg/m^2^/day of hydrocortisone equivalent) provided some improvement but caused significant hyperglycemia. Dexamethasone 0.02 mg twice daily mixed with insulin injections was trialed but caused discomfort without significant improvement in the reactions.

In an effort to bridge to pump therapy, inpatient insulin desensitization was performed with cetirizine, ranitidine, and montelukast pre-medication. Lispro was incrementally increased, starting with smaller doses through an IV pump, followed by larger doses through the Tandem T:Slim pump. Despite desensitization, localized reactions persisted. Colchicine 0.3 mg twice daily was trialed but was unsuccessful in decreasing reactions. Subcutaneous lispro and twice daily detemir injections were resumed upon discharge.

Additional immunotherapies including weekly methotrexate, monthly IVIG (1 g/kg), and triannual rituximab were initiated, but localized reactions continue. Despite a TDD of 2.26 units/kg/day, HbA1c was 9.5%. She was also followed for hypercholesterolemia and BMI above the 97th percentile. She was approved for a pancreas transplant 6 years after reactions first developed. At the time of writing, she had received her transplant 6 weeks prior. She was receiving 10 units of Levemir daily (TDD of 0.15 units/kg/day), which is being weaned off, and she has not required rapid-acting insulin.

## Case C

Patient C is an 8-year-old boy, diagnosed with T1D at age 14 months. He developed an eczematous localized type IV reaction to lispro 1.8 years after diagnosis (**Figure 1E**). He trialed all available insulins, but localized reactions recurred 6–8 h after injections. Glucoses fluctuated from 50 to 450 mg/dL despite adherence to the recommended basal bolus insulin regimen. He also trialed insulin pump therapy with lispro and aspart but developed firm nodules and persistent hyperglycemia. Localized erythematous, nodular reactions persisted despite topical glucocorticoids and local phototherapy.

He underwent extensive testing with allergy/immunology, dermatology, and genetics. A skin biopsy 2 years after initial reactions demonstrated perivascular and interstitial dermatitis with inflammatory cell infiltrate containing lymphocytes and histiocytes. Percutaneous skin prick testing was negative for cresols, glulisine, glargine, lispro, and aspart. Patch testing was negative to these allergens, but positive to nickel and acrylates, which are found in pump infusion sets (8). Intradermal skin testing was performed, with a 1:100 dilution for insulins, zinc, and cresol and 1:20 dilution for glycerin. This was positive at 24 h for all insulins including aspart, lispro, degludec, glargine, and regular insulin, but negative for additives. Insulin IgG antibody was 2.33 nmol/mL (from 0.09 nmol/mL at diabetes diagnosis) and insulin IgE was negative. Whole exome sequencing was negative for FOXP3 mutation, involved in regulatory T-cell development (9). IgG, IgA, C3, C4, and lymphocyte counts were normal.

He was started on methotrexate and up titrated to 13.5 mg/m^2^. He continued aspart and glargine injections. The localized eczematous-like reactions improved. However, rare cutaneous injection site erythema 12–24 h after injections persisted, partly relieved by topical triamcinolone. Upon resuming pump therapy, he had recurrence of erythema and small nodules on days 2–3 of pump site wear, though less extensive than previously reported. Three years after starting methotrexate, he started to develop more frequent local reactions and methotrexate was titrated up to 18 mg/m^2^/week. Dupilumab was trialed for 4 months without improvement. At the time of writing, HbA1c was 8.3% on a TDD 0.67 units/kg/day. The older brother was diagnosed with T1D at 18 months and has had no insulin reactions to date.

## Case D

Patient D is a 15-year-old male adolescent, diagnosed with T1D at age 2 years. He tolerated glulisine for 6 years, but upon transitioning to insulin aspart, the patient developed erythematous, firm, tender nodules presenting several hours after injection and lasting several days (**Figure 1F**). Upon resuming glulisine, he developed similar reactions. He trialed all available insulins, pump therapy with a steel and Teflon cannula, and a trial of desensitization, but reactions persisted. Delivery of steroids mixed with insulin via pump was ineffective. Oral dexamethasone slightly improved symptoms, but exacerbated hyperglycemia. Treatments with cyclosporine, IVIG, dupilumab, and dapsone led to adverse events, including malaise, emesis, and aseptic meningitis, without improvement in the injection site reactions. Additionally, topical tacrolimus and cromolyn were ineffective. The family considered traveling to Europe to pursue the Accu-Chek® DiaPort, which delivers insulin via a percutaneous port with intraperitoneal catheter (6). However, they ultimately refrained since it was not FDA approved in the US.

Despite receiving 1.7 units/kg/day of aspart via an Omnipod pump, with site changes every 36–48 h, HbA1c remains elevated at 9.0% at the time of writing.

## Review of the literature

### Overview of hypersensitivity reactions

Type I, III, and IV insulin hypersensitivity reactions have been reported. Although immediate, type I, IgE-mediated insulin hypersensitivity reactions are most common, type III and type IV delayed reactions can also occur (10). Immediate reactions occur within 24 h. Delayed reactions may arise within 12 h, or up to 48–72 h later (1, 2).

### Causative agents in insulin hypersensitivity

Insulin reactions were more common with non-purified bovine or porcine insulins, but can occur with recombinant human insulins. The prevalence of reactions to insulin products among all ages ranges between 0.1% and 3%; less than one-third are due to insulin itself and most are attributed to additives (1, 2, 10).

While type I and III reactions are often due to insulin itself, type IV more commonly results from additives (1, 2) (**Table 2**) including cresols, protamine, glycerin, phenol, and/or zinc (11). Meta-cresol is common to all insulins; glycerin and phenols are common to most. Protamine is complexed to intermediate-acting insulins to delay absorption (1, 2).

**Table 2:** Insulin types, additives, and hypersensitivity reactions.

Insulin duration, hypersensitivity type	Insulin	Metacresol	Protamine	Glycerin	Phenol	Zinc	Polysorbate 20	Other Additives
Rapid acting	Aspart	x		x	x	x		Dibasic sodium (Na) phosphate, Na chloride, Na hydroxide, hydrochloric acid
Rapid acting	Lispro	x		x	x	x		Dibasic Na phosphate, hydrochloric acid, Na hydroxide
Rapid acting	Glulisine	x					x	Na chloride, tromethamine, hydrochloric acid, Na hydroxide
Short acting	Regular	x		x		x		Hydrochloric acid, Na hydroxide
Intermediate acting	Neutral Protamine Hagedorn (NPH)	x	x	x	x	x		Dibasic Na phosphate, hydrochloric acid, Na hydroxide
Intermediate acting	U-500 concentrated	x		x		x		Hydrochloric acid, Na hydroxide
Intermediate mixture	75% lispro protamine, 25% lispro	x	x	x	x	x		Dibasic Na phosphate, hydrochloric acid, Na hydroxide
Intermediate mixture	70% aspart protamine, 30% aspart	x	x	x	x	x		Dibasic Na phosphate, hydrochloric acid, Na hydroxide
Intermediate mixture	NPH, regular	x	x	x	x	x		Dibasic Na phosphate, hydrochloric acid, Na hydroxide
Intermediate to long acting	Detemir	x		x	x	x		Dibasic Na phosphate
Long acting	Glargine	x		x		x	x	Hydrochloric acid, Na hydroxide
Long acting	Degludec	x		x	x	x		Hydrochloric acid, Na hydroxide
Hypersensitivity:								
Type I hypersensitivity	x							
Type IV hypersensitivity		x	x	x	x	x	x	x

Alternate allergen hypotheses exist, such as yeast or bacteria used in synthesizing recombinant insulin or analogues. Epitope alteration or protein misfolding during synthesis and purification may also mediate reactions (10). Conversely, crystallization has been hypothesized to mask the antigenicity of insulin molecules. Crystallized zinc human insulin was successfully used in a patient without zinc allergy who had reacted to a de-crystallized form (12).

Reactions to acrylates and other components of insulin pump infusion sets have been reported (8, 13). Fundamentally, infusion cannulas are composed of either Teflon (plastic polymers) or stainless steel (metals) (13). These reactions may be overcome by changing the infusion set material or using multiple daily injection therapy.

### Type I insulin hypersensitivity

Type I, IgE-mediated reactions most commonly present within seconds to minutes but can occur after several hours (1, 2). Symptoms include localized urticaria, though life-threatening reactions can also occur. Anaphylaxis can present as bronchospasm, lip swelling, hypotension, and/or severe gastrointestinal symptoms (**Supplementary Data Sheet 1**) (14); intramuscular epinephrine is the first treatment (15).

**Figure 2:**
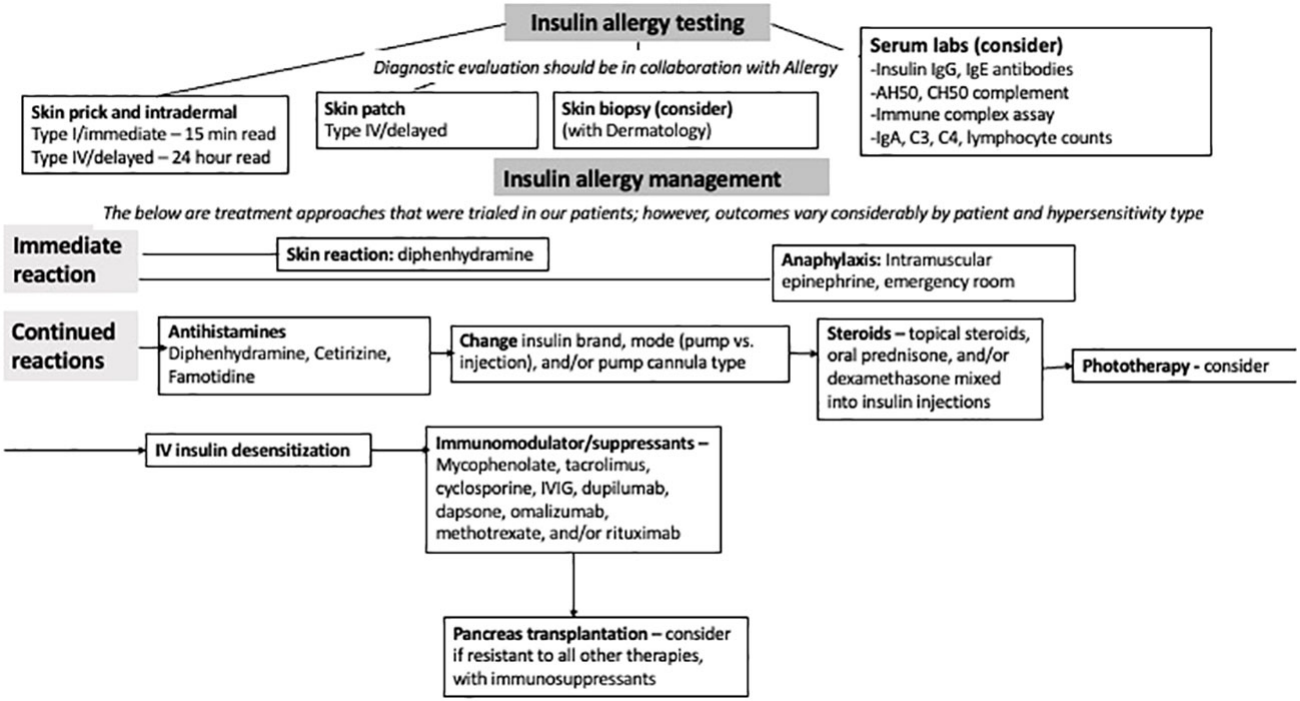
Flowchart of insulin allergy testing and management.

There are several diagnostic options. Skin prick testing entails exposure to a drop of potential allergen, then gently pricking the skin with a needle (**Figure 2**). The appearance of an erythematous, pruritic wheal within 15 min suggests sensitization (16). Skin intradermal testing is more sensitive, but lacks specificity, as up to 40%–50% of diabetes patients with no clinical signs of insulin allergy may have positive results (5). All patients on insulin may have detectable titers of IgE against insulin (17). Conversely, IgE levels may appear low due to consumption during acute hypersensitivity episodes (10). Double-blinded, placebo-controlled graded drug challenges remain the gold standard for type I insulin allergy confirmation. There have been associations between certain HLA haplotypes (-DR4 and -DR7) and IgG antibodies to insulin, whereas HLA-DR3 may be protective (12).

Avoidance and desensitization are commonly used for type I hypersensitivity reactions. By gradually exposing patients to increasing continuous insulin infusion doses over hours to days, desensitization aims to induce tolerance by depleting basophils and mast cells, inhibiting IgG antibodies, and stimulating suppressive T-cells (3). Certain protocols have been successful in published cases (18). However, despite a history of type I reactions in patient A, desensitization was unsuccessful.

In two 9-year-old boys with generalized urticarial insulin reactions, IV regular insulin was infused to achieve normoglycemia before slowly decreasing the infusion rate while simultaneously increasing the rate of subcutaneous insulin lispro infusion in a 1:1 ratio. After reaching an appropriate basal insulin rate, meal boluses and corrections were initiated beginning with a square wave bolus over 3 h before advancing to more rapid rates of infusion. By 48 h, normal boluses were tolerated without reactions. In a follow-up 15 months later, both children remain on CSII and oral antihistamines, with only occasional urticaria (18).

**Figure 3:**
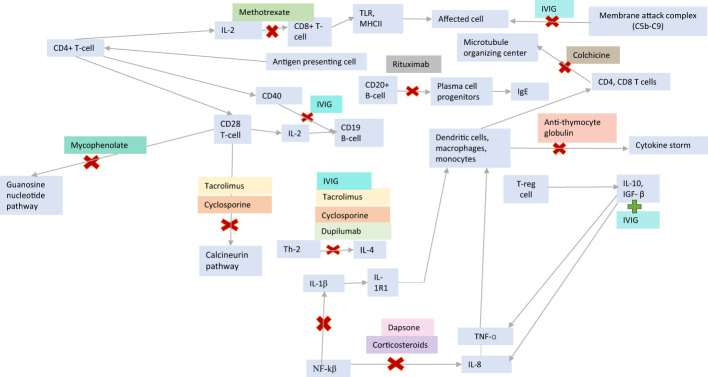
Insulin hypersensitivity treatments and immunologic mechanisms of actions.

Various immunosuppressants (19) were trialed in our patients with type I reactions (**Figure 3**). Although not used on patient A as it contains polysorbate 20, omalizumab, an anti-IgE recombinant monoclonal antibody, has been helpful in adult cases (4, 20). In a 50-year-old woman with T1D and urticarial reactions, omalizumab was initially not used due to an elevated IgE level of 3,710 IU/mL. Rituximab was used at a weekly dose of 375 mg per square meter of body surface area for four doses, followed by mycophenolate mofetil until IgE decreased to 675 IU/mL. Then, omalizumab was initiated, leading to marked improvement of reactions. The patient was started on prednisolone 2 mg daily, and 9 months later, reactions had not recurred (4).

### Type III insulin hypersensitivity

Type III hypersensitivity reactions are mediated by IgG or IgM antigen–antibody immune complexes (16, 21). These complexes deposit in the tissue and lead to tissue damage through complement system activation, chemotactic agent release, neutrophil presence, and inflammation (21). These reactions most often present as indurated, painful subcutaneous nodules, but can be subclassified as localized Arthus reactions, generalized serum sickness, or serum sickness-like reactions (1, 22). Arthus reactions can present as indurated, painful subcutaneous nodules with edema and/or hemorrhage at the local injection or pump site (1). Serum sickness can arise when immune complexes target vessel walls and tissues, presenting as fever, vasculitis, urticarial rash, and polyarthralgia (22).

In patient B, rituximab, which reduces B-cells and IgE (4), and colchicine, which mediates tubulin dysfunction and decreases IL-1, IL-6, and TNF (23, 24), were used. Methotrexate, corticosteroids, and IVIG were used in several of our patients with type III and IV reactions. Methotrexate acts by apoptosis of alloreactive T cells and inhibition of purine synthesis enzymes (25, 26), while corticosteroids inhibit IL-1β, TNF-α, and NK-Kβ (25). Although the exact mechanism of IVIG is unknown, it is thought to involve inhibition of monocytes and macrophages, induction of TGF-β and IL-10, inhibition of antigen-presenting B-cells, IL-4, CD40, and formation of the C5b–C9 membrane-attack complex (27).

In one case, a 26-year-old woman with T1D developed erythematous nodules after 3 years of pump use (22). Despite trialing various treatments, she was admitted 1 month later in diabetic ketoacidosis and serum sickness-like reaction, including fever and polyarthralgia. Intravenous insulin was started while plasmapheresis with fresh frozen plasma every 1–3 days initially improved the reaction. While awaiting pancreas transplantation, she developed a fatal anaphylactic reaction during plasmapheresis (22).

### Type IV insulin hypersensitivity

Type IV, T-cell-mediated delayed insulin hypersensitivity reactions commonly present as erythematous, eczematous-like patches and plaques (26).

Skin patch testing can be used for diagnosis by applying a potential allergen and covering it with a dressing. The patches are removed at 48 h and the site is checked for any reactions and re-examined at 96 h (16). Additionally, skin biopsy entails obtaining a small skin sample for microscopic examination (28). Various immunologic findings may be seen, including spongiotic dermatitis and perivascular lymphocytic infiltrate (26).

In patient A, anti-thymocyte globulin, which induces T- and B-cell apoptosis and dendritic cell interference, led to transient improvements in reactions. Mycophenolate, which depletes guanosine in T- and B-cells, was also used. Cyclosporine, dupilumab, and dapsone were ineffective in decreasing localized reactions in patient D. Cyclosporine and tacrolimus are calcineurin inhibitors that prevent T-cell, IL-2, and IL-4 activation. Dupilumab blocks IL-4 and IL-13 responses by inhibiting IL-4Rα (27). Dapsone inhibits T cells, IL-8, TNF-α, and IL-1β (29–31).

### Pancreas transplantation

Pancreas transplantation has been a treatment of last resort for adults with insulin hypersensitivity but is rarely used in children (32). The donor portal vein is anastomosed to the recipient inferior vena cava or iliac vein. Secretions are drained through the bladder (20% of cases), or a duodenojejunostomy or duodenoduodenostomy (80%) (33, 34). In patient A, reactions were eliminated post-transplant and euglycemia was maintained without exogenous insulin. Adult pancreas-alone-transplant graft survival rates are 69%–84% at 3 years and 53% at 5 years. Pancreas availability is limited as donors <30 years with excellent organ function and low BMI are preferred (34, 35). As with any transplant, there remains a potential for rejection and return of insulin deficiency (33). Long-term immunosuppression risks include infection and malignancy (34, 35). Patients must understand that transplantation is akin to trading one disease process for another.

## Conclusion

Although rare, given the life-sustaining nature of insulin in T1D, it is crucial to learn from these cases of insulin hypersensitivity. Successful treatment remains elusive despite trials with various insulins, antihistamines, and even pancreas transplantation. Further studies to elucidate the underlying pathophysiology and development of targeted treatments are needed.

## Data availability statement

The original contributions presented in the study are included in the article/**Supplementary Material**. Further inquiries can be directed to the corresponding author.

## Ethics statement

Institutional review board approval for publication was obtained per institutional guidelines for case series that involve more than three patients. Ethics guidelines were met in accordance with The Code of Ethics of the World Medical Association (Declaration of Helsinki). Written informed consent was obtained from the patient’s parents/legal guardians for publication of the details of this medical case and any accompanying images, and patient assent was obtained for patients aged >12 years old.

## Author contributions

EHA authored this manuscript in close collaboration with BEM. All authors reviewed the manuscript prior to submission. EHA, JBG, AMA, MDB, JPB, and BEM were involved in the clinical care of patient A; EMJ and MDB were involved in the clinical care of patient B; MK, KPM, and BIP were involved in the clinical care of patient C; and KSB and TLP were involved in the clinical care of patient D.
