# Supplementary material for: Case Report: Insulin hypersensitivity in youth with type 1 diabetes
Source: Front Endocrinol (Lausanne). 2023 Oct 20;14:1226231. doi: 10.3389/fendo.2023.1226231 (PMC10624121; doi:10.3389/fendo.2023.1226231)
Supplement: Supplementary file 1 [file DataSheet_1.docx]

Supplementary Material

Case Report: Insulin hypersensitivity in youth with type 1 diabetes

**Supplementary Data Sheet 1** – Anaphylaxis criteria according to 2006 National Institute of Allergy and Infectious Disease and the Food Allergy and Anaphylaxis Network (NIAID/FAAN) diagnostic criteria^36^

Anaphylaxis is highly likely when any one of the following 3 criteria are fulfilled:

1. Acute onset of an illness (minutes to several hours) with involvement of the skin, mucosal tissue, or both (e.g., generalized hives, pruritus or flushing, swollen lips-tongue-uvula) AND AT LEAST ONE OF THE FOLLOWING:
2. Respiratory compromise (e.g. dyspnea, wheeze-bronchospasm, stridor, reduced peak expiratory flow, hypoxemia)
3. Reduced blood pressure or associated symptoms of end-organ dysfunction (e.g. hypotonia [collapse], syncope, incontinence)
4. Two or more of the following that occur rapidly after exposure to a likely allergen for that patient (minutes to several hours):
   1. Involvement of the skin-mucosal tissue (e.g. generalized hives, itch-flush, swollen lips-tongue-uvula)
   2. Respiratory compromise (e.g. dyspnea, wheeze-bronchospasm, stridor, reduced peak expiratory flow, hypoxemia)
   3. Reduced blood pressure or associated symptoms (e.g. hypotonia [collapse], syncope, incontinence)
   4. Persistent gastrointestinal symptoms (e.g. crampy abdominal pain, vomiting)
5. Reduced BP after exposure to known allergen for that patient (minutes to several hours):
   1. Infants and children: low systolic BP (age specific) or greater than 30% decrease in systolic BP†
   2. Adults: systolic BP of less than 90 mm Hg or greater than 30% decrease from that person’s baseline

*†Low systolic blood pressure for children is defined as less than 70 mm Hg from 1 month to 1 year, less than (70 mm Hg + [2 x age]) from 1 to 10 years, and less than 90 mm Hg from 11 to 17 years***
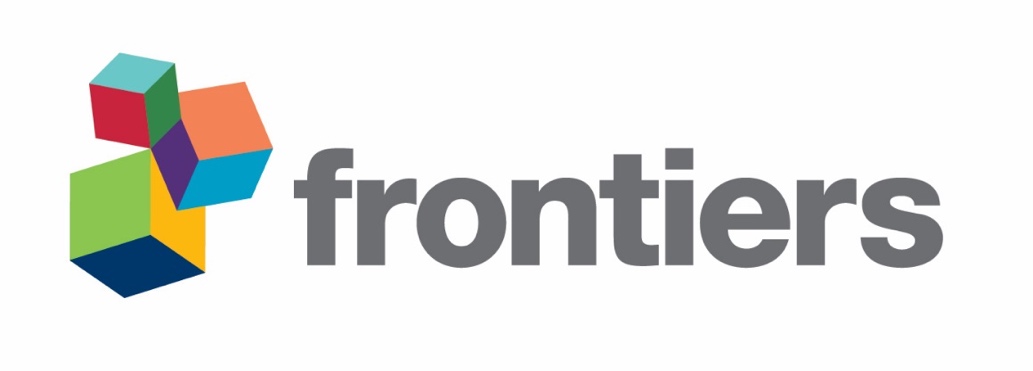
**
